# Correlation of ^18^F-sodium fluoride uptake and radiodensity in extraosseous metastases of medullary thyroid carcinoma

**DOI:** 10.20945/2359-4292-2023-0152

**Published:** 2024-04-11

**Authors:** Cristina Emiko Ueda, Laís Flausino Dias, Camila de Godoi Carneiro, Marcelo Tatit Sapienza, Carlos Alberto Buchpiguel, Paulo Schiavom Duarte

**Affiliations:** 1 Instituto do Câncer de São Paulo Divisão de Medicina Nuclear São Paulo SP Brasil Divisão de Medicina Nuclear, Instituto do Câncer de São Paulo (Icesp), São Paulo, SP, Brasil; 2 Universidade de São Paulo Faculdade de Medicina Departamento de Radiologia e Oncologia São Paulo SP Brasil Divisão de Medicina Nuclear, Departamento de Radiologia e Oncologia, Faculdade de Medicina, Universidade de São Paulo (FMUSP), São Paulo, SP, Brasil

**Keywords:** Medullary thyroid carcinoma, calcification, 18F-NaF, PET/CT, radiodensity, Hounsfield unit

## Abstract

**Objective:**

Although ^18^F-sodium fluoride (^18^F-NaF) uptake is frequently observed in extraosseous metastases of medullary thyroid carcinoma (MTC) with calcification, it can also occur in metastatic sites without visible calcium deposition, leading to the hypothesis that visually undetectable calcium accumulation may be responsible for this uptake. The aim of this study was to indirectly support this hypothesis by analyzing the correlation between the degree of ^18^F-NaF uptake and radiodensity in extraosseous MTC metastases, since calcium deposition can increase attenuation even when not visually detectable.

**Subjects and methods:**

Extraosseous metastatic lesions of 15 patients with MTC were evaluated using ^18^F-NaF positron-emission tomography (PET)/computed tomography (CT) and segmented by levels of standardized uptake value (SUV). The correlation between mean SUV and mean Hounsfield unit (HU) values was assessed for the entire group of segments and for two subgroups with different mean HU values.

**Results:**

Very high correlations were observed between mean SUV and mean HU values for both the entire group of segments and the subgroup with a mean HU value greater than 130 (p = 0.92 and p = 0.95, respectively; p < 0.01). High correlation (*p* = 0.71) was also observed in the subgroup with mean HU values ranging from 20 to 130 (p < 0.01).

**Conclusion:**

The findings of the present study suggest that there is an association between ^18^F-NaF uptake and calcium deposition in extraosseous metastases of MTC, supporting the hypothesis that visually undetectable calcium accumulation may be responsible for ^18^F-NaF uptake in regions without visible calcium deposition.

## INTRODUCTION

Extraosseous metastatic sites of medullary thyroid carcinoma (MTC) have been shown to uptake ^18^F-sodium fluoride (^18^F-NaF), as reported in previous studies using positron emission tomography (PET) (1,2). The underlying mechanism responsible for this uptake has not been extensively studied in the literature, but it is thought to be related to the calcification process occurring within the tumor, particularly in regions of amyloid deposition (3,4). However, there have been instances in which uptake has been observed in metastatic regions without macroscopic calcium deposition, which could be attributed to visually undetectable calcium accumulation. Indeed, in some cases, macroscopic calcification has been observed in follow-up studies of brain (5) and liver (6) metastatic lesions of MTC that initially presented with ^18^F-NaF uptake but no gross calcification on computed tomography (CT) images. Furthermore, in evaluations of atherosclerotic plaque, ^18^F-NaF uptake has been linked to the ongoing process of calcium deposition (7–9).

In this line of reasoning, Irkle and cols. used immunohistochemical analysis of carotid endarterectomy specimens to demonstrate a strong correlation between the ^18^F-NaF signal and alizarin red (calcification marker) staining, indicating that ^18^F-NaF specifically colocalizes with vascular calcification (10). The authors also used autoradiography/histology and μPET/μCT scanning to analyze carotid specimens and found that while microcalcifications identified on histology were not detectable by μCT, they were identified by an increased ^18^F-NaF μPET signal.

Based on these previous findings, we hypothesized that the radiodensity of distinct regions of extraosseous metastases of MTC and the degree of ^18^F-NaF uptake in these regions are correlated, as both parameters are associated with calcification. Therefore, we analyzed in the present study the correlation between these two parameters in extraosseous metastatic MTC lesions.

## SUBJECTS AND METHODS

### Patients

We retrospectively evaluated 15 patients with MTC who had undergone ^18^F-NaF PET/CT scans to assess bone metastatic disease. These patients were selected among 31 patients with MTC who had undergone ^18^F-NaF PET/CT scans at our institution. Of these, 16 were excluded from the analysis as they did not have soft tissue metastatic disease or their soft tissue metastases could not be adequately characterized on low-dose CT images without intravenous contrast. The 15 patients included in the analysis had extraosseous metastases that were visible on the low-dose CT images of the PET/CT scans.

In addition to the PET/CT analysis, we obtained the following information from the patients’ records: age at PET/CT scanning, sex, MTC type, age at thyroidectomy, TNM staging at diagnosis, and treatments prior to the PET/CT scan.

The study was conducted at the Nuclear Medicine Section of the *Instituto do Câncer do Estado de São Paulo* (Icesp) and was approved by the ethical committee of the *Hospital das Clínicas da Universidade de São Paulo* (CAAE: 01905518.6.0000.0065). All patients were followed up at ICESP, and the requirement to obtain informed consent was waived.

### ^18^F-NaF PET/CT studies

The ^18^F-NaF PET/CT studies were acquired using a Discovery PET/CT 690 with time-of-flight technology (GE Healthcare, Waukesha, WI, USA). The patients received an injection of approximately 185 MBq of ^18^F-NaF. Around 45–60 minutes after the injection, all patients underwent whole-body (vertex to toes) three-dimensional PET/CT acquisition. The emission images were obtained at 1 minute per bed position (15 cm axial scan field of view with 3 cm of overlapping), with 13 to 15 bed positions per study. The CT transmission images (30 mAs) were obtained for attenuation correction without intravenous contrast. Other CT acquisition parameters were voltage 120 kVp, rotation time 0.5 second, pitch 1.375, and axial slice thickness 3.75 mm. The PET image reconstruction was performed using an iterative technique (ordered subset expectation maximization [OSEM]) with 2 iterations and 24 subsets for all studies. The CT images were reconstructed with the conventional filtered back projection (FBP) method using Bone Plus reconstruction filter (GE Healthcare). In patients with more than one ^18^F-NaF PET/CT study, the last study was analyzed.

### Image analysis

A nuclear medicine physician with 20 years of experience used the software LIFEx (11) to define the contours of the most prominent extraosseous metastatic lesions observed in the ^18^F-NaF PET/CT study. The contours formed volumes of interest (VOIs), which were merged into a single main VOI for patients with multiple VOIs. However, for patients with widespread lung (1 patient) or liver (4 patients) metastatic disease, the VOIs were drawn around the entire organs instead of the metastases contours due to a difficulty in defining the boundaries between the lesions and the tissue around them.

The main VOIs of the 15 patients were then segmented based on levels of standardized uptake value (SUV) thresholds, resulting in a total of 128 derived VOIs, and mean SUV and mean Hounsfield unit (HU) values were calculated within each VOI. The SUV levels used to segment the VOIs varied depending on the size and range of the SUV within the lesions.

Further, VOIs with mean HU below 20 (*i.e.*, values under extraosseous radiodensity) (12) were excluded as they could contain a significant amount of fat or air regions. This resulted in a final number of 95 derived VOIs analyzed in 13 patients (two patients did not present any VOI with mean HU value ≥ 20). These VOIs were divided into two subgroups based on mean HU value, with one group ranging from 20 to 130 (n = 58) and the other with mean HU value > 130 (n = 37). This threshold was chosen based on a value frequently used to establish extraosseous calcification in the analysis of atherosclerotic coronary disease (13,14). This separation was performed to evaluate whether the two parameters (HU and SUV) were associated in segments with and without this criterion of calcification.

### Statistical analysis

The null hypothesis that the data exhibited normal distribution was tested using the Kolmogorov-Smirnov test. Since this test rejected the null hypothesis of normal distribution, we used Spearman's nonparametric correlation test to assess the association between the two parameters (HU and SUV) in all 95 segmented VOIs and in the two subgroups divided by HU threshold (mean HU ranging from 20 to 130 and mean HU > 130) using the software SPSS, version 20.0 (IBM, Armonk, NY, USA).

## RESULTS

The characteristics of the patients are presented in Table 1. Ten patients were female and five were male. At the PET/CT study, their median age was 40 years (range 17-78 years), and their median calcitonin level was 19,064 pg/mL (range 108-134,802 pg/mL). Ten patients had sporadic MTC, three had multiple endocrine neoplasia (MEN) type 2A, and two had MEN type 2B. The median age at thyroidectomy was 26 years (range 12-70 years). Thirteen patients had been treated before the PET/CT procedure. The treatments varied from radiotherapy to target therapies and are detailed in Table 1, along with TNM stage at diagnosis.

**Table 1:** Characteristics of the patients included in the study.

ID	Sex	Age (years)	MTC type	Age at thyroidectomy (years)	TNM staging at diagnosis	Treatments before PET/CT study	Calcitonin level at PET/CT study (pg/mL)	Soft tissue metastatic sites analyzed	SUVmax in the main VOI	Main VOI (mL)	Number of derived VOIs	Number of derived VOIs ≥ 20 HU
1	F	40	MEN 2A	22	T2N1bM1	Sor/Van	112,357	Liver and mediastinal lymph nodes	9.3	1,328.0	9	9
2	F	74	Sporadic	50	TxNxM1	ZA/BR	11,525	Cervical and mediastinal lymph nodes	4.6	8.8	4	3
3	F	38	MEN 2A	22	T2N1bM1	None	424	Lung nodule and hilar lymph node	9.5	1.0	2	1
4	F	34	Sporadic	33	T2N1bM1	Van	12,560	Mediastinal mass	3.8	174.0	3	2
5	M	19	MEN 2B	12	T3N1bM1	CR/Iri/Tha/177Lu/Van	39,650	Lung	6.1	1,631.0	6	0
6	M	44	MEN 2A	30	TXN1M1	None	134,802	Liver	11.8	1,750.0	10	9
7	F	17	MEN 2B	14	T4N1bM1	Van	61,765	Liver	7.4	951.6	6	6
8	M	24	Sporadic	16	T1N1bMx	CR/Cab/Van	108	Cervical and hilar lymph nodes	5.4	18.1	5	5
9	F	35	Sporadic	21	T4N1bMx	CR/Cab	19,064	Mediastinal and hilar lymph nodes	15.1	15.4	11	1
10	F	68	Sporadic	42	Missing	CR/Che	18,804	Cervical lymph nodes	2.6	8.2	3	3
11	M	49	Sporadic	46	TxN1 M1	CR/Dac/Cap/Van	108,069	Cervical, mediastinal, and hilar lymph nodes	5.2	177.8	5	1
12	F	78	Sporadic	70	T3N1M0	CR/ZA/Dac/Cap	19,015	Cervical/mediastinal mass	35.9	130.3	19	18
13	F	43	Sporadic	18	TxN1M1	Sor	43,268	Liver	5.9	1,785.0	5	3
14	F	38	Sporadic	35	T4N1bM1	ZA / BR / Sor / Che	12391	Liver	62.8	1,640.0	37	34
15	M	65	Sporadic	57	T4aN1Mx	Sun / Dac / Paz	67958	Thoracic lymph nodes	3.2	74.30	3	0

Abbreviations: ≥20 HU, mean value of derived VOI above or equal to 20 HU; ^177^Lu, 177Lu-dotatate; BR, bone radiotherapy; Cab, cabozantinib; Cap, capecitabine; Che, chemotherapy (not specified); CR, cervical radiotherapy; Dac, dacarbazine; F, female; HU, Hounsfield unit; ID, patient identification number; Iri, irinotecan; M, male; MEN, multiple endocrine neoplasia; MTC, medullary thyroid carcinoma; None, no treatment given other than thyroidectomy before PET/CT scanning; Paz, pazopanib; Sor, sorafenib; Sun, sunitinib; SUVmax, maximum standardized uptake value; Tha, thalidomide; Van, vandetanib; VOI, volume of interest; ZA, zoledronic acid.

**Figure 1:**
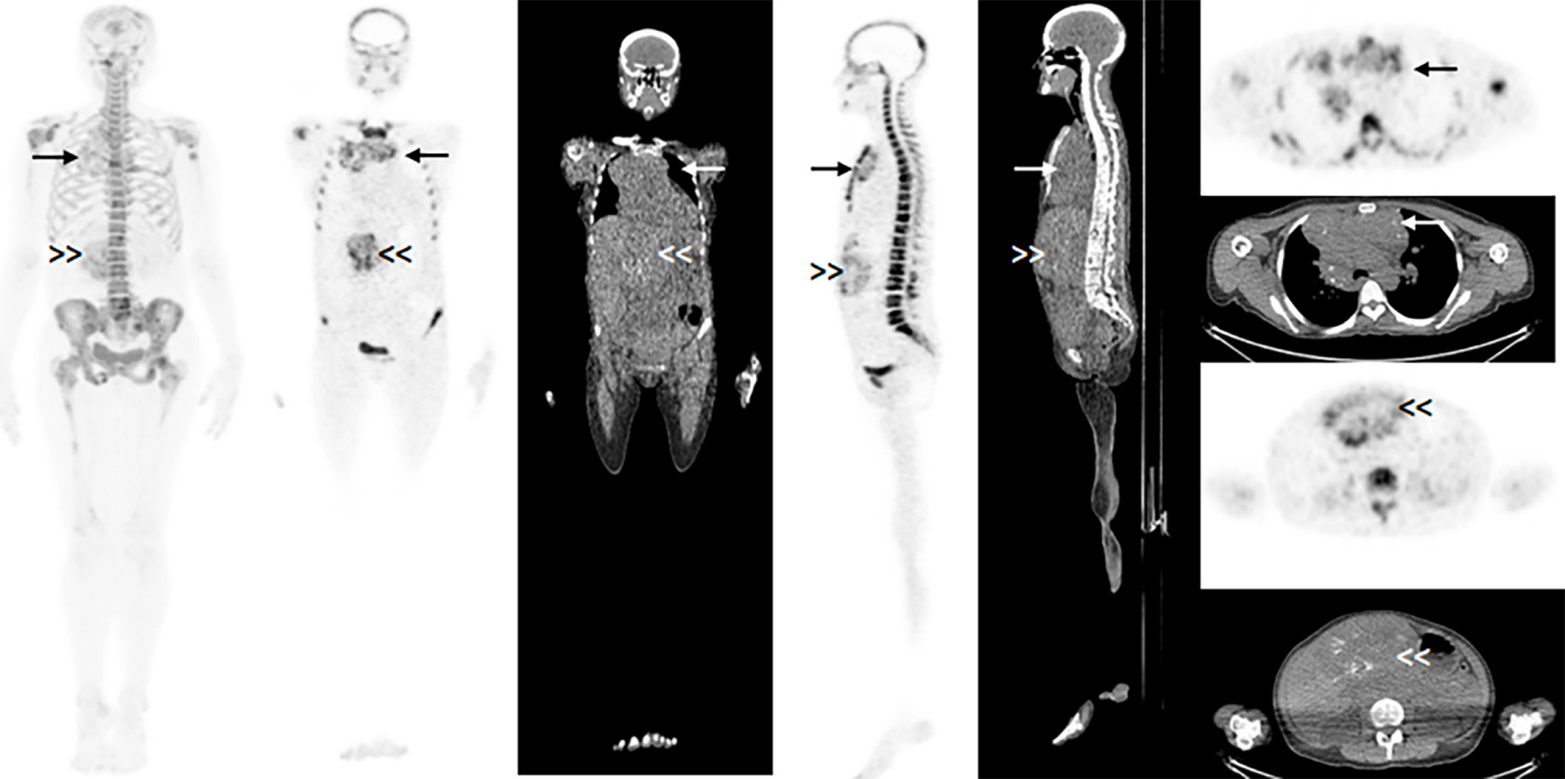
^18^F-NaF PET/CT study showing multiple bone metastatic lesions and extraosseous disease in the liver (double arrowhead) and mediastinum (arrow). Maximal intensity projection (MIP) coronal image (on the left side of the figure) and coronal, sagittal, and axial images of the metabolic and anatomical parts of the study at the level of the extraosseous lesions.

**Figure 2:**
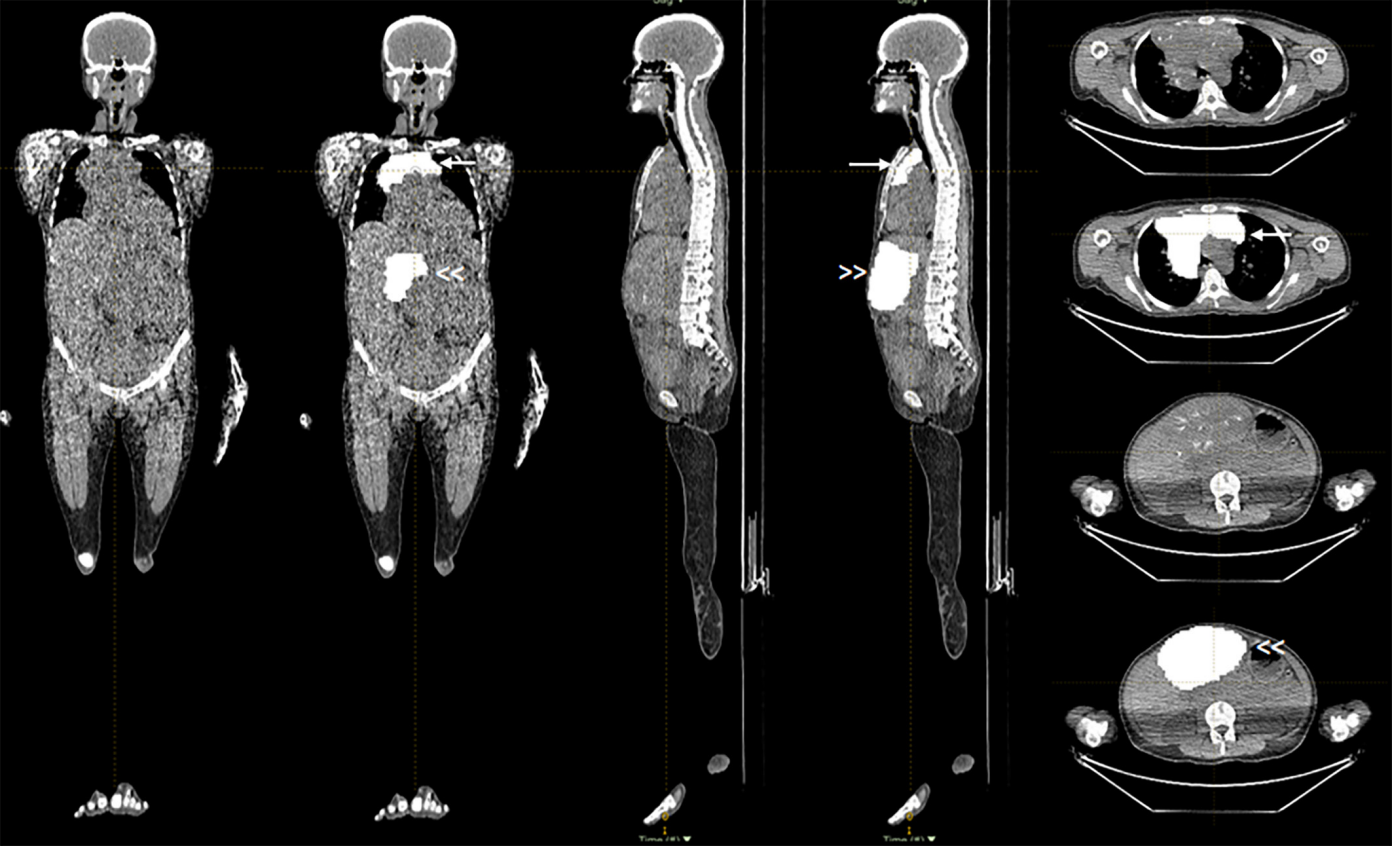
Coronal, sagittal, and axial images of the CT component of the ^18^F-NaF PET/CT study showing the extraosseous lesions without and with the VOIs at the level of the mediastinum (arrow) and liver (double arrowhead).

**Figure 3:**
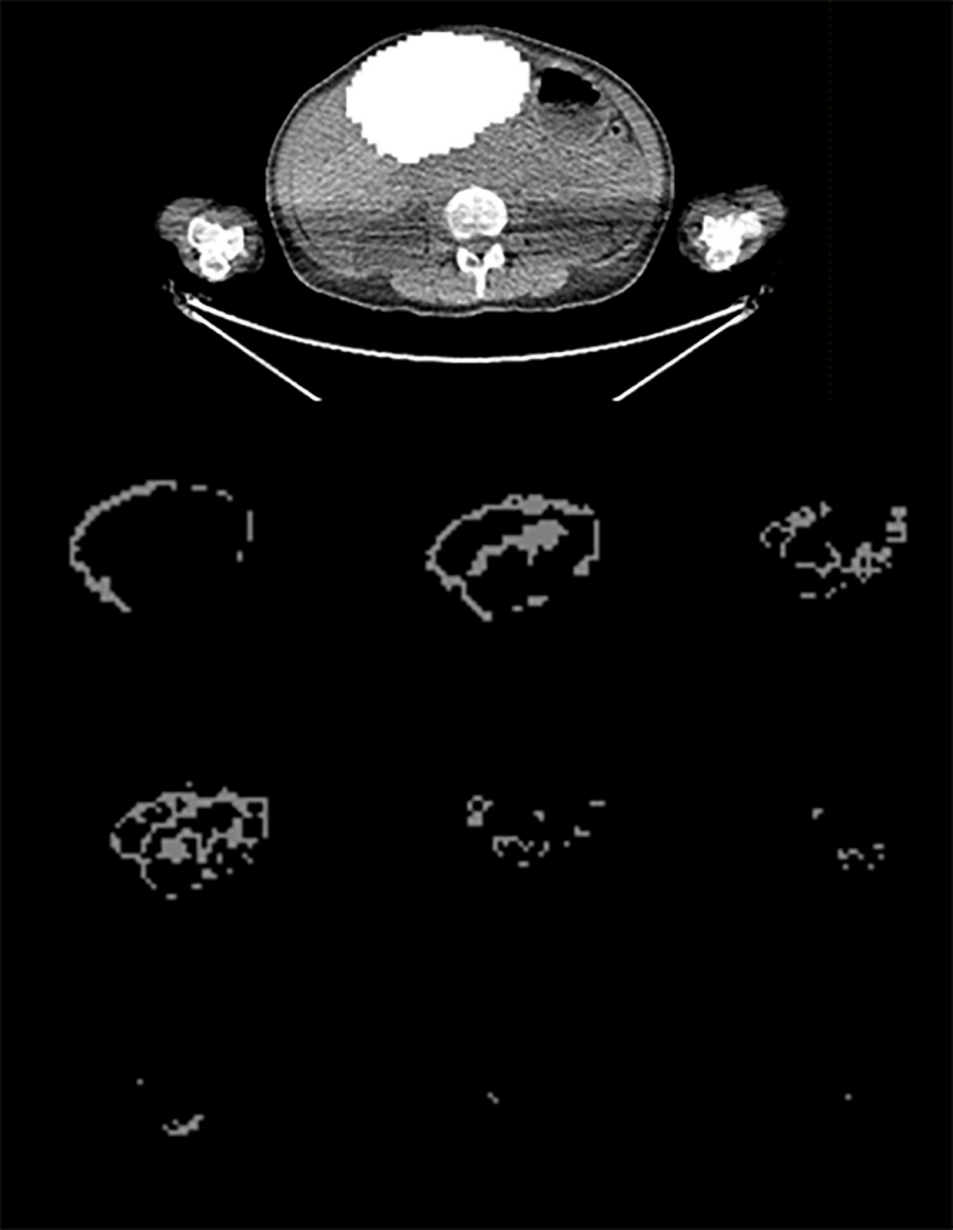
Axial CT image showing a slice of the main VOI at the level of the liver (top image) and the nine derived VOIs (bottom images) based on the SUV thresholds for the patient presented in Figures 1 and 2.

The median value for the maximum SUV (SUVmax) was 6.1 (range 2.6-62.8). The median value for the main VOI was 174.0 mL (range 1.0-1785.0 mL). The total number of derived VOIs was 128, and the number of VOIs per patient varied from 2 to 37. The total number of derived VOIs with a mean HU value ≥ 20 was 95, and the number of VOIs per patient varied from 0 to 34. Figures 1 and 2 show an example of a patient with multiple bone and extraosseous metastatic lesions and of the VOIs contouring in the soft tissue lesions. Figure 3 illustrates the main VOI and the nine derived VOIs based on SUV thresholds for the patient presented in Figures 1 and 2.

**Figure 4:**
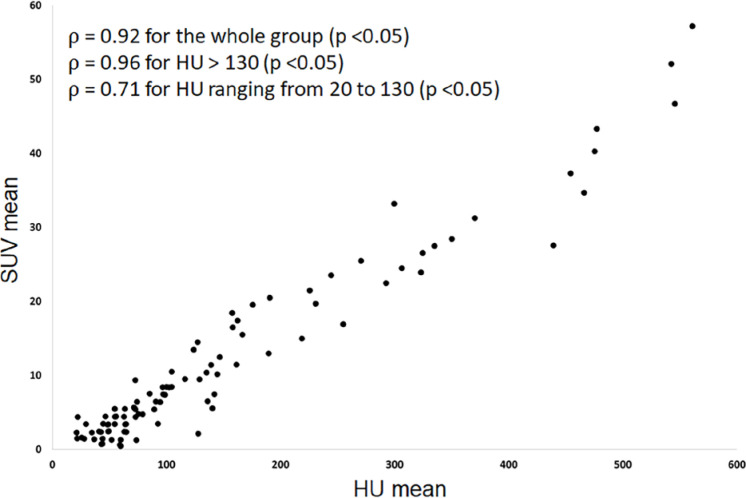
Graph showing the relation between mean HU values (Y axis) and mean SUV (X axis) in the 95 derived VOIs. There is a very high correlation between these two variables for the whole group of VOIs and for the ones with HU values above 130. There is also a high correlation for the VOIs with HU values less than or equal to 130.

The correlation analysis revealed very high levels of correlations for both the entire group of 95 derived VOIs and the subgroup with a mean HU > 130 (p = 0.92 and ρ = 0.96, respectively, p < 0.05) (Figure 4). A high correlation level (p = 0.71) was also observed in the subgroup with mean HU ranging from 20 to 130 (p < 0.05) (Figure 4), suggesting a progressive association between ^18^F-NaF uptake and calcium deposition even before the establishment of the calcification process by a commonly used standard criterion (13,14).

The results of two patients contributed to more than half of the derived VOIs with mean HU value ≥ 20 (one contributed with 18 derived VOIs and the other with 34 derived VOIs). Due to that, we performed subanalyses excluding these patients, which showed that if the data of any of these two patients were excluded, p remained above 0.8 (p < 0.01) for the entire group of remaining VOIs, and if both patients were excluded simultaneously, p was 0.68 (p < 0.01). Therefore, the correlation between HU and SUV values was not exclusively due to the derived VOIs from a few patients but to the whole group of individuals.

## DISCUSSION

Previous PET/CT studies have demonstrated that ^18^F-NaF can detect bone metastases (2,15) and sporadic extraosseous metastases (1,6) of MTC, sometimes with greater accuracy than ^68^Ga-labeled somatostatin analogues (2,16). The underlying mechanism of ^18^F-NaF uptake by MTC extraosseous metastases is not fully understood but is believed to be due to the progressive calcification of amyloid deposits that frequently occur in these tumors (4). However, other mechanisms may also be involved, as the uptake of ^18^F-NaF by extraosseous metastases of other tumors, including breast cancer (17), melanoma (18), and lung cancer (19), has also been reported. Regardless of the underlying cause, ^18^F-NaF uptake appears to be present even before macroscopic foci of calcifications are detected in the extraosseous lesions, and the strong positive correlation observed between SUV and HU values in this analysis supports the hypothesis of an association between the calcification process, characterized by the progressive increase in the radiodensity of the different parts of the lesion, and the radiopharmaceutical uptake. Importantly, this correlation was observed for regions with both high HU levels (>130) and low HU levels (≤130), suggesting that even in areas with low calcium deposits, there is already progressive ^18^F-NaF uptake.

However, in the current discussion, it is imperative to elucidate certain points. First, even though this analysis was conducted with a limited number of patients, it is plausible that the pathophysiologic mechanism prevailing in the extraosseous metastatic sites is represented in at least a subset of patients with MTC. Nonetheless, future investigations should aim to increase the sample size, but we anticipate some potential difficulties in conducting such an analysis owing to the rarity of MTC (20) and the scarcity of cases with extraosseous metastatic sites that can be appropriately segmented within a single medical center. Therefore, multicenter studies involving a larger number of MTC patients would be indispensable to accurately evaluate the correlation between HU and SUV in ^18^F-NaF PET/CT. Second, it is crucial to address the rationale behind the exclusion of derived VOIs with HU values of less than 20. As mentioned in the Introduction, these segments could contain a substantial amount of fat or air regions derived from sampling surrounding normal lung or fat tissues instead of metastatic tissues. This could occur either intentionally, as in cases where patients presented with disseminated pulmonary metastatic disease and the VOIs were drawn around the whole lungs, or unintentionally, when the boundaries of the lesion were not correctly depicted at the moment of the VOI definition. These sampling errors of normal tissues, associated with small discrepancies between the anatomic and metabolic images – especially in the thorax due to breathing, which is common in PET/CT studies (21) – may cause a false increment of the SUVs in normal regions. Therefore, as the HU and SUV in these segments could potentially confound the overall results, we excluded them from the analysis. This exclusion resulted in the elimination of 33 derived VOIs from 11 patients, with five of these patients having all or almost all VOIs excluded (Table 1). Most of the metastatic sites in these five patients were in the lungs or close to the lungs. Third, it is important to clarify that the range of SUV intervals used to create the derived VOIs varied from patient to patient and were subjectively defined by the nuclear medicine physician based on the size and the maximum SUV in the VOIs. Typically, this interval was one SUV unit. However, for VOIs with a high maximum SUV, the difference in SUV interval between the derived VOIs was increased to two or three units. Additionally, if an interval of one unit generated derived VOIs that were too small (less than 10 voxels), the range of the interval was also increased to enlarge the size of the derived VOI. Fourth, in the cases of patients who had undergone multiple ^18^F-NaF PET/CT studies, it is imperative to justify the selection of the latest study for the analysis. This is due to the fact that the latest study is likely to display a greater number of calcium deposits, resulting from a longer ongoing calcification process, as well as a more heterogeneous calcification pattern with varying degrees of calcium deposits within the metastatic lesion. Consequently, the lesions observed in the latest ^18^F-NaF PET/CT study would more probably reflect the association between radiopharmaceutical uptake and radiodensity. Fifth, it is impossible to guarantee that all the calcification processes observed in the soft tissue metastatic lesions resulted from the natural evolution of the disease and not from the response of the metastatic site to therapy, since calcification can be related to chemotherapy or chemoembolization (22). However, even if the calcification associated with therapy is present, it should not be the main process responsible for all calcifications in extraosseous metastatic sites observed in patients with MTC, since we do not see the same frequency and intensity of calcification in the follow-up of extraosseous metastatic lesions of other tumors also submitted to chemotherapy. Moreover, although some portions of calcification could be attributed to therapy rather than disease progression, this does not alter the main finding of our analysis, which is a robust correlation between ^18^F-NaF uptake and radiodensity. Last, the results obtained from this analysis may have important diagnostic implications. This is because the uptake of ^18^F-NaF in metastatic sites, even prior to the emergence of visible calcifications, could prove to be useful in identifying small lesions that may be missed during CT scans. As such, when interpreting ^18^F-NaF PET/CT scans in patients with MTC, it is crucial for nuclear medicine physicians to actively search for areas of uptake outside of the bone that could potentially represent metastatic disease. Additionally, these findings may have therapeutic implications since the uptake of ^18^F-NaF in both bone and extraosseous metastatic sites could provide an opportunity to explore the use of ^223^radium as a therapeutic agent in a select group of patients with MTC, similar to the potential theranostic use of radiolabeled somatostatin analogues and prostate-specific membrane antigen (PSMA) (23).
